# Prognostic value of PD‐L1 expression in combination with CD8^+^ TILs density in patients with surgically resected non‐small cell lung cancer

**DOI:** 10.1002/cam4.1243

**Published:** 2017-11-23

**Authors:** Hui Yang, Jinpeng Shi, Dongmei Lin, Xuefei Li, Chao Zhao, Qi Wang, Limin Zhang, Tao Jiang, Sha Zhao, Xiaozhen Liu, Yijun Jia, Yajun Zhang, Weijing Cai, Caicun Zhou

**Affiliations:** ^1^ Department of Medical Oncology Shanghai Pulmonary Hospital & Thoracic Cancer Institute Tongji University School of Medicine Shanghai 200433 China; ^2^ Key Laboratory of Carcinogenesis and Translational Research (Ministry of Education) Peking University Cancer Hospital and Institute Beijing 100142 China; ^3^ Department of Lung Cancer and Immunology Shanghai Pulmonary Hospital & Thoracic Cancer Institute Tongji University School of Medicine Shanghai 200433 China; ^4^ Department of Pathology Yancheng Third People's Hospital Yancheng 224001 China; ^5^ Department of Thoracic Surgery Yancheng Third People's Hospital Yancheng 224001 China

**Keywords:** CD8, non‐small cell lung cancer, prognosis, programmed cell death ligand‐1, tumor infiltrating lymphocytes

## Abstract

To investigate the prognostic value of PD‐L1 expression combined with CD8^+^ TILs density in patients with resected NSCLC and correlations with clinicopathological features. We retrospectively enrolled 178 patients with resected NSCLC from 2011 to 2015. All surgical primary and 58 matched metastatic lymph node specimens were tested for PD‐L1, CD8^+^ TILs, and oncogenic alterations. PD‐L1^+^ was detected in 71 (39.9%) and CD8^high^ TILs in 74 (41.6%) cases. Smoking, SqCC, and *EGFR* ^−^ were associated with both PD‐L1^+^ and CD8^high^ TILs. Patients with CD8^high^ TILs had longer OS (*P *=* *0.012). PD‐L1^−^ was significantly associated with longer OS in patients with oncogenic alterations (*P *=* *0.047). By multivariate analysis, CD8^high^ TILs (HR = 0.411; 95% CI, 0.177–0.954; *P *=* *0.038), rather than PD‐L1, was the independent predictive factor for OS. The longest and shortest OS were achieved in patients with PD‐L1^+^/CD8^high^ and PD‐L1^+^/CD8^low^, respectively (*P *=* *0.025). Inconsistent PD‐L1 expression levels were observed in 23 of 58 (39.7%) patients with primary and matched metastatic lymph node specimens. Of them, CD8^high^ TILs was significantly associated with longer OS in patients with metastatic lymph nodes and/or consistent PD‐L1 expression (*P *=* *0.017 and 0.049, respectively). The combination of PD‐L1 and CD8^+^ TILs density, instead of PD‐L1 alone, suggested impressive prognostic values in NSCLC patients. Less than half of patients with resected NSCLC experienced inconsistent PD‐L1 expression between primary and metastatic lesions. The level of PD‐L1 expression in advanced NSCLC needs to be evaluated more comprehensively.

## Introduction

Lung cancer is one of the most common malignancies and the leading cause of worldwide cancer mortality 1. Limitations (such as drug resistance) have emerged in traditional strategies, including chemotherapy and molecular‐targeted therapy. Since the approval of pembrolizumab as a first‐line therapy in non‐small cell lung cancer (NSCLC) therapy, increased attention has been paid to the study of immune checkpoints 2. Currently, the markers of interest are the CD8^+^ tumor infiltrating lymphocytes (TILs) density and the expression levels of tumor programmed cell death ligand‐1 (PD‐L1) 3.

Programmed death receptor‐1 (PD‐1) is a protein receptor of the CD28 family expressed on the surface of T, B, and natural killer (NK) cells that regulates their activation and proliferation. Its ligand, PD‐L1, is frequently overexpressed in many kinds of human malignancies. The binding of PD‐L1 to PD‐1 induces apoptosis or exhaustion in activated T cells and limits the effector function of T cells in peripheral tissues during inflammatory responses 4. Blockade of PD‐1/PD‐L1 pathway has been shown to enhance the antitumor effector functions in the tumor microenvironment including T‐cells activity 5, 6. Anti‐PD‐1/PD‐L1 antibodies, such as nivolumab, pembrolizumab, and atezolizumab have demonstrated promising and amazing efficacy against various tumors in several clinical trials, especially those involving NSCLC 7, 8, 9, 10. Preliminary studies of NSCLC patients indicate that clinical benefits to immune checkpoint inhibitors are associated with elevated PD‐L1 expression levels on tumor cells and increased TILs numbers 9, 11. Moreover, Teng and colleagues proposed that four different types of tumor microenvironment exist based on the presence or absence of TILs and PD‐L1 expression levels (type I: TILs^+^ and PD‐L1^+^; type II: TILs^−^ and PD‐L1^−^; type III: TILs^+^ and PD‐L1^−^; type IV: TILs^−^ and PD‐L1^+^) 12. Different types of tumor microenvironment show distinct responses to PD‐1/PD‐L1 antibodies. Type I tumors could gain the greatest benefit from PD‐1/PD‐L1 antibodies treatment 12. This suggested that both PD‐L1 expression and TILs density may play a critical role in tumor microenvironment and immune checkpoint therapy. However, the prognostic value of PD‐L1 expression and CD8^+^ TILs density in Chinese NSCLC patients remains unclear. To date, few studies focusing on this issue have been published, and the case number of those studies is small 13. Furthermore, those studies evaluated samples only obtained by tumor biopsies, which were too small for assessment of the location and orientation of CD8^+^ TILs. Their results concerning the correlation between PD‐L1 expression/CD8^+^ TILs and prognosis in NSCLC are inconsistent 14, 15, 16.

Hence, we performed this study that aimed to investigate the prevalence and prognostic value of PD‐L1 expression and CD8^+^ TILs density in Chinese patients with surgically resected NSCLC. We also analyzed the correlation between clinicopathological characteristics and PD‐L1 expression/CD8^+^ TILs density in NSCLC.

## Methods

### Study patients

Patients who were diagnosed with NSCLC and underwent surgical resection at Yancheng Third People's Hospital and Shanghai Pulmonary Hospital from 2011 to 2015 were evaluated. Patients with missing baseline clinicopathological characteristics and follow‐up data were excluded. Of the remaining patients, those who had formalin‐fixed and paraffin‐embedded (FFPE) specimens representing primary lesions containing adequate tumor cells and T cells were enrolled in this study. Patients who were staged as p‐TxN1‐3Mx without FFPE specimens representing metastatic lymph nodes were excluded. Finally, 178 total eligible patients were included. This study was conducted in accordance with the Declaration of Helsinki and was approved by the Ethics Committee and Institutional Review Board (IRB) of Yancheng Third People's Hospital and Shanghai Pulmonary Hospital. A written informed consent was obtained from each participant in order to use the clinical data for research.

### Immunohistochemistry

Immunohistochemistry was performed according to the protocol recorded in our previous study 17. In this study, anti‐human PD‐L1 (#13684, clone E1L3N, Cell Signaling Technology, Danvers, MA, diluted 1:200) and CD8 monoclonal antibodies (#M7103, clone C8144B, DAKO, Glostrup, Denmark, diluted 1:200) were used as primary antibodies, and a peroxidase‐labeled secondary antibody (REAL EnVision Detection Reagent Peroxidase Rabbit/Mouse, DAKO, Glostrup, Denmark) was applied to visualize the antigen. PD‐L1 expression was defined as the percentage of tumor cells displaying membranous immunoreactivity, either in the central or marginal tumor region; and PD‐L1^−/+^ was determined by cut‐off value of 5%, which was based on several previous clinical trials of anti‐PD‐1/PD‐L1 drugs in NSCLC; 11, 18, 19 among PD‐L1^+^ cases, we defined PD‐L1 strong positivity (PD‐L1^high^) as PD‐L1 > 50% (Fig. S1). Lymphocytes with cytoplasmic expression of CD8 infiltrating within tumor region, either in the central or marginal tumor region, were defined as CD8^+^ TILs (Fig. S1). Based on the percentage of CD8^+^ TILs presented within tumor region, we stipulated CD8^low/high^ TILs with cut‐off value of 5%, which was determined in a manner similar to that of a previous publication 13. Moreover, breast cancer cell line MDA‐MB‐231 and placenta tissues were used as the positive controls for PD‐L1 IHC‐staining, and breast cancer cell line MCF‐7 was used as the negative control for PD‐L1 IHC‐staining (Fig. S2). All IHC analyses were evaluated by two experienced pathologists by means of manual quantification through their naked eyes, and the mean value of the determinations was used for further analyses. The Pearson's correlation coefficient between the two pathologists for CD8^+^ TILs as a continuous variable was as follows: *r *=* *0.79; *P *<* *0.001. Neither pathologists were aware of the identity of the specimens.

### ODM analyses

All oncogenic driver mutation (ODM) analyses were performed at Thoracic Cancer Institute, Tongji University School of Medicine (Shanghai, China). *EGFR*,* KRAS*,* ALK*,* ROS1*,* HER2*, and *RET* status were detected as described in our previous studies 20, 21, 22, 23. All the ODM analyses were identified by the commercially available AmoyDx^®^ Gene Mutation Detection Kits (AmoyDx Co. Ltd., Xiamen, China). To express ODM concisely, ODM^+^ refers to any one or more oncogenic driver mutations and ODM^−^ refers to pan‐negative for the six above‐mentioned oncogenic diver mutations.

### Statistical analyses

Correlations between PD‐L1 expression/CD8^+^ TILs density and patients' clinicopathological characteristics were performed with Chi‐square test for categorical variables and ANOVA and Tukey's multiple comparison tests for continuous variables. The Kaplan–Meier method was used for survival analyses, and the significance of differences between groups was evaluated by the log‐rank test. The Cox proportional hazards model was used for univariate and multivariate analyses to calculate the hazard ratios (HR) and 95% confidence intervals (95% CI). Overall survival (OS) was calculated from the date of diagnosis to death from any cause or was censored at the last follow‐up date. *P* values were considered statistically significant if <0.05 (two‐sided). All statistical analyses were performed by SPSS for Windows (version 17.0; IBM Corporation, Armonk, NY, USA).

## Results

### Clinicopathological characteristics

A total of 178 patients were included. The median age at diagnosis was 62 years (range: 24–77 years). One‐hundred thirteen (63.5%) patients were male, and 103 (57.9%) patients had a history of smoking. The majority of histological types was adenocarcinoma (ADC, 137/178), including five lepidic, 18 papillary, 81 acinar, 29 solid, three enteric and one minimally invasive ADC (IMA), which was followed by 41 squamous cell carcinomas (SqCCs). Of them, 85 (47.8%), 47 (26.4%), 39 (21.9%; IIIA, 37; IIIB, 2), and seven (3.9%) patients had stages I–IV diseases at the time of diagnosis. With respect to ODM status, *EGFR*,* KRAS*,* HER2*,* RET*,* ALK*, and *ROS1* alterations were detected in 73, 16, five, four, three, and three cases, respectively (co‐alterations were detected in six cases). We found that PD‐L1^high^ was more likely to be detected in patients with SqCC (*P *=* *0.001), *EGFR* wild type (*EGFR* ^−^) (*P *=* *0.045), and ODM^−^ (*P *=* *0.046) (Table 1).

**Table 1:** Baseline clinicopathological characteristics and molecular alterations of included patients (n = 178)

	Total	PD‐L1 > 5%	PD‐L1 ≤ 5%	*P*	PD‐L1 > 50%	PD‐L1 ≤ 50%	*P*	CD8 high	CD8 low	*P*
Age										
<65	110	43	67	0.782	26	84	0.836	48	62	0.477
≥65	68	28	40		17	51		26	42	
Sex										
Male	113	54	59	**0.005**	32	81	0.087	48	65	0.747
Female	65	17	48		11	54		26	39	
Smoking										
Never	75	20	55	**0.002**	14	61	0.144	24	51	**0.027**
Current/former	103	51	52		29	74		50	53	
Histology										
ADC	137	46	91	**0.002**	24	109	**0.001**	51	86	**0.031**
Solid	29	18	11		12	17		14	15	
Lepidic	10	5	5		5	5		2	3	
Papillary	18	5	13		3	15		9	9	
Acinar	77	21	56		9	68		22	55	
Others	8	2	6		1	7		4	4	
SqCC	41	25	16		19	26		23	18	
p‐Stage										
I	85	33	52	0.782	21	64	0.870	43	42	**0.020**
II–IV	93	38	55		22	71		31	62	
I–II	132	51	81	0.564	34	98	0.398	60	72	0.075
III–IV	46	20	26		9	37		14	32	
*EGFR*										
Wild type	105	52	53	**0.002**	31	74	**0.045**	52	53	**0.010**
Mutant type	73	19	54		12	61		22	51	
*KRAS*										
Wild type	162	61	101	0.053	38	124	0.487	64	98	0.075
Mutant type	16	10	6		5	11		10	6	
ODM										
Wild type	80	40	40	**0.013**	25	55	**0.046**	38	42	0.147
Mutant type	98	31	67		18	80		36	62	

PD‐L1, programmed cell death ligand‐1; ADC, adenocarcinoma; SqCC, squamous cell carcinoma; *EGFR, epidermal growth factor receptor; KRAS, Kirsten rat sarcoma viral oncogene*; ODM, oncogenic driver mutations.

### The correlation between PD‐L1 Expression/CD8^+^ TILs density and OS among the patients

PD‐L1^+^ was detected in 71 (39.9%) patients, and it was significantly associated with male gender (*P *=* *0.005), current/former smokers (*P *=* *0.002), SqCC (*P *=* *0.002), and *EGFR* ^−^ (*P *=* *0.002), but not with age (*P *=* *0.782), p‐stage (*P *=* *0.782), or *KRAS* mutation status (*P *=* *0.053). We restricted our analyses to CD8^+^ TILs due to tissue availability, which is generally thought to be the population mainly benefitting from treatment with PD‐1/PD‐L1 antibodies 24. CD8^high^ TILs were detected in 74 (41.6%) cases and were significantly associated with smoking (*P *=* *0.027), SqCC (*P *=* *0.031), p‐stage I (*P *=* *0.020) and *EGFR* ^−^ (*P *=* *0.010). In addition, it was observed that CD8^high^ was associated with PD‐L1^+^/PD‐L1^high^, and CD8^low^ was associated with PD‐L1^−^ (*P *<* *0.001) (Table 1).

**Figure 1:**
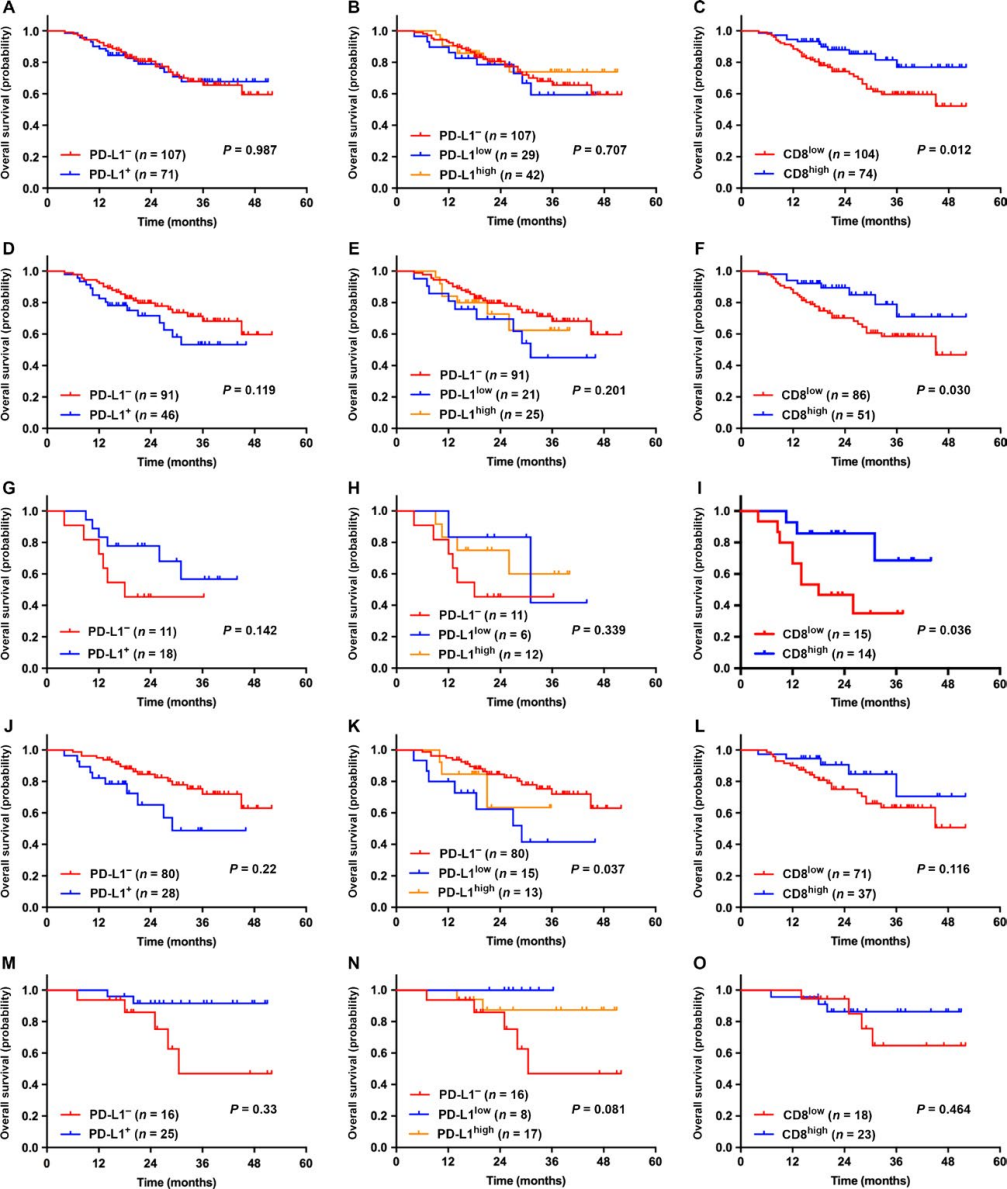
**The correlation between the expression levels of PD‐L1 (on tumor cells) or CD8^+^ TILs density and OS among NSCLC patients**. CD8^high^ TILs was associated with better OS, but the expression levels of PD‐L1 showed no correlation with OS among NSCLC patients (A–C), patients with ADC (D–F), and solid ADC (G–I), respectively; in patients with SqCC (M–O), PD‐L1^+^ was associated with longer OS, but CD8^+^ TILs density showed no correlation with OS; on the contrary, in patients with non‐solid ADC (J–L), PD‐L1^–^ may suggest favorable OS, and patients with PD‐L1^low^ reached the longest OS, but CD8^+^ TILs density showed no correlation with OS.

**Figure 2:**
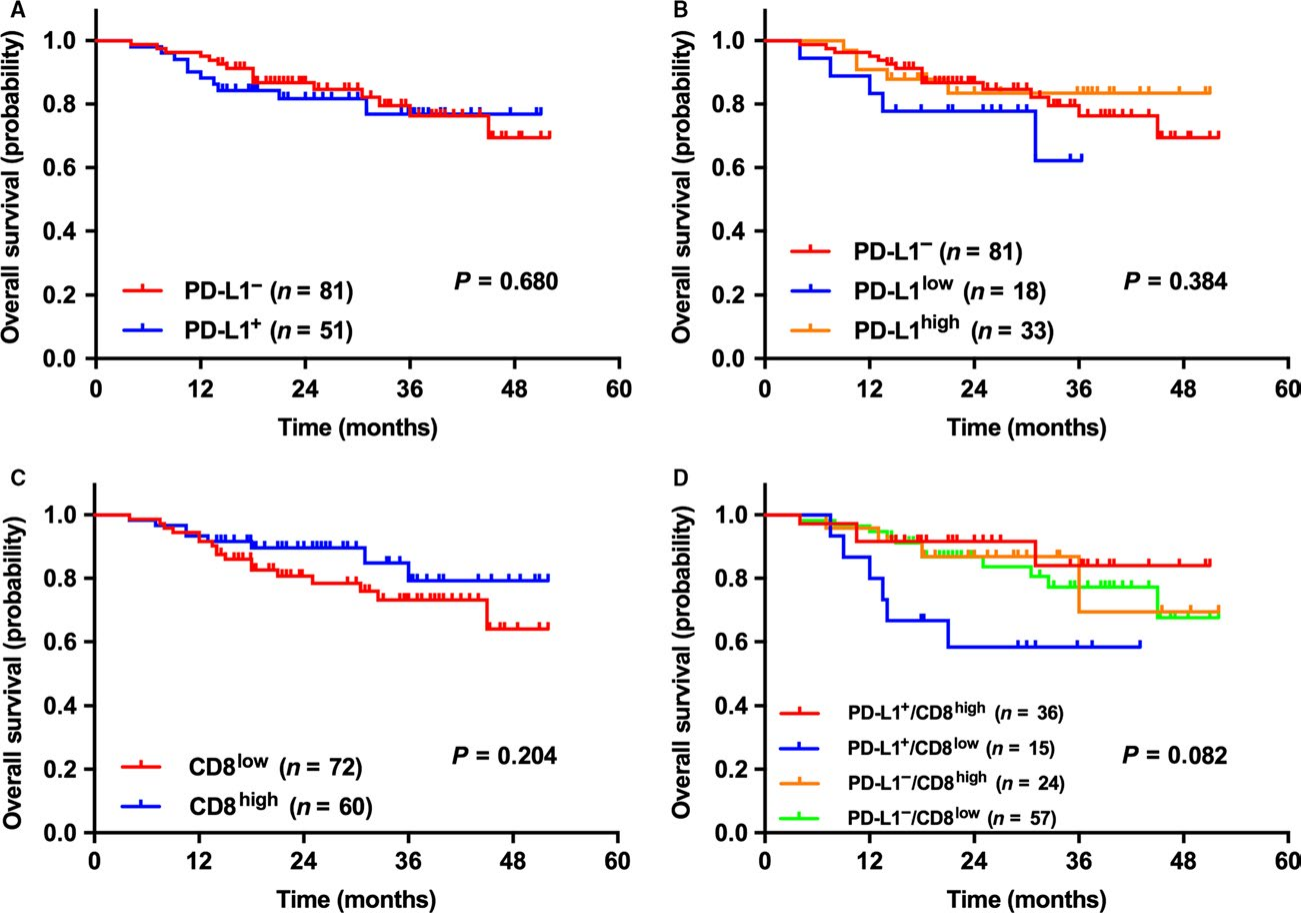
**Survival analysis of p‐stage I/II NSCLC patients based on the expression levels of PD‐L1 (on tumor cells), CD8^+^ TILs density and the combination of PD‐L1 expression (5% cut‐off) and CD8^+^ TILs density (5% cut‐off).** As for p‐stage I/II NSCLC patients, the expression levels of either PD‐L1 or CD8 was not associated with OS, not even the combination of both.

Furthermore, we analyzed the correlation between PD‐L1 expression/CD8^+^ TILs density and OS. The median follow‐up time was 27 months (range, 6–55 months). Among the entire cohort, patients with CD8^high^ TILs had longer OS (*P *=* *0.012), but there was no statistical difference between PD‐L1^−/+^ and OS (*P *=* *0.987). In patients with ADC and solid ADC, we obtained similar results in which CD8^high^ TILs were associated with better OS (*P *=* *0.030 & 0.036, respectively), and PD‐L1^−/+^ showed no correlation with OS (*P *=* *0.119 & 0.142, respectively). Interestingly, in patients with non‐solid ADC and SqCC, PD‐L1^+^ was associated with more satisfactory OS than PD‐L1^−^ (*P *=* *0.022 & 0.033, respectively), however, CD8^+^ TILs density did not correlate with OS (*P *=* *0.116 & 0.464, respectively). When we sub‐grouped PD‐L1^low/high^ in PD‐L1^+^ cases for survival analyses, statistically significant difference was only achieved in patients with non‐solid ADC (*P *=* *0.037) (Fig. 1). The prognostic value of PD‐L1 expression and CD8^+^ TILs density in p‐stages I/II NSCLC patients was also investigated. In this cohort, no prognostic value of the two parameters was verified (*P *=* *0.680 & 0.204, respectively), not even both combined (*P *=* *0.082) (Fig. 2).

Among the whole cohort, univariate and multivariate analyses were performed. Multivariate analyses revealed that p‐stages II‐IV (vs. p‐stage I; HR = 2.105; 95% CI, 1.036–4.279; *P *=* *0.040) and CD8^high^ TILs (vs. CD8^low^ TILs; HR = 0.411; 95% CI, 0.177–0.954; *P *=* *0.038) were independent and significant predictive factors for OS in NSCLC instead of PD‐L1 expression (HR = 1.682; 95% CI, 0.833–3.397; *P *=* *0.147) (Table 2).

**Table 2:** Univariate and multivariate analyses of clinicopathological factors associated with OS

	Univariate			Multivariate		
	HR	95% CI	*P* value	HR	95% CI	*P* value
Age						
<65	1	0.954–3.393	0.070	1.684	0.880–3.222	0.115
≥65	1.799					
Sex						
Male	1	0.467–1.658	0.692			
Female	0.880					
Smoking						
Never	1	0.863–3.076	0.132			
Current/former	1.630					
Histology						
Solid	1	0.260–1.025	0.059	0.587	0.283–1.215	0.151
Non‐solid	0.516					
p‐Stage						
I	1	1.345–5.290	**0.005**	2.105	1.036–4.279	**0.040**
II–IV	2.667					
PD‐L1						
Negative	1	0.871–3.143	0.124	1.682	0.833–3.397	0.147
Positive	1.655					
CD8						
Low	1	0.199–0.943	**0.035**	0.411	0.177–0.954	**0.038**
High	0.433					
*EGFR*						
Wild type	1	0.509–1.791	0.886			
Mutant type	0.955					
*KRAS*						
Wild type	1	0.267–2.129	0.593			
Mutant type	0.754					
ODM						
Wild type	1	0.413–1.461	0.434			
Mutant type	0.777					

OS, overall survival; HR, hazard ratio; CI, confidence interval; PD‐L1, programmed cell death ligand‐1; *EGFR, epidermal growth factor receptor; KRAS, Kirsten rat sarcoma viral oncogene;* ODM, oncogenic driver mutations.

### The correlation between combination of PD‐L1 expression & CD8^+^ TILs density and OS among the patients

**Figure 3:**
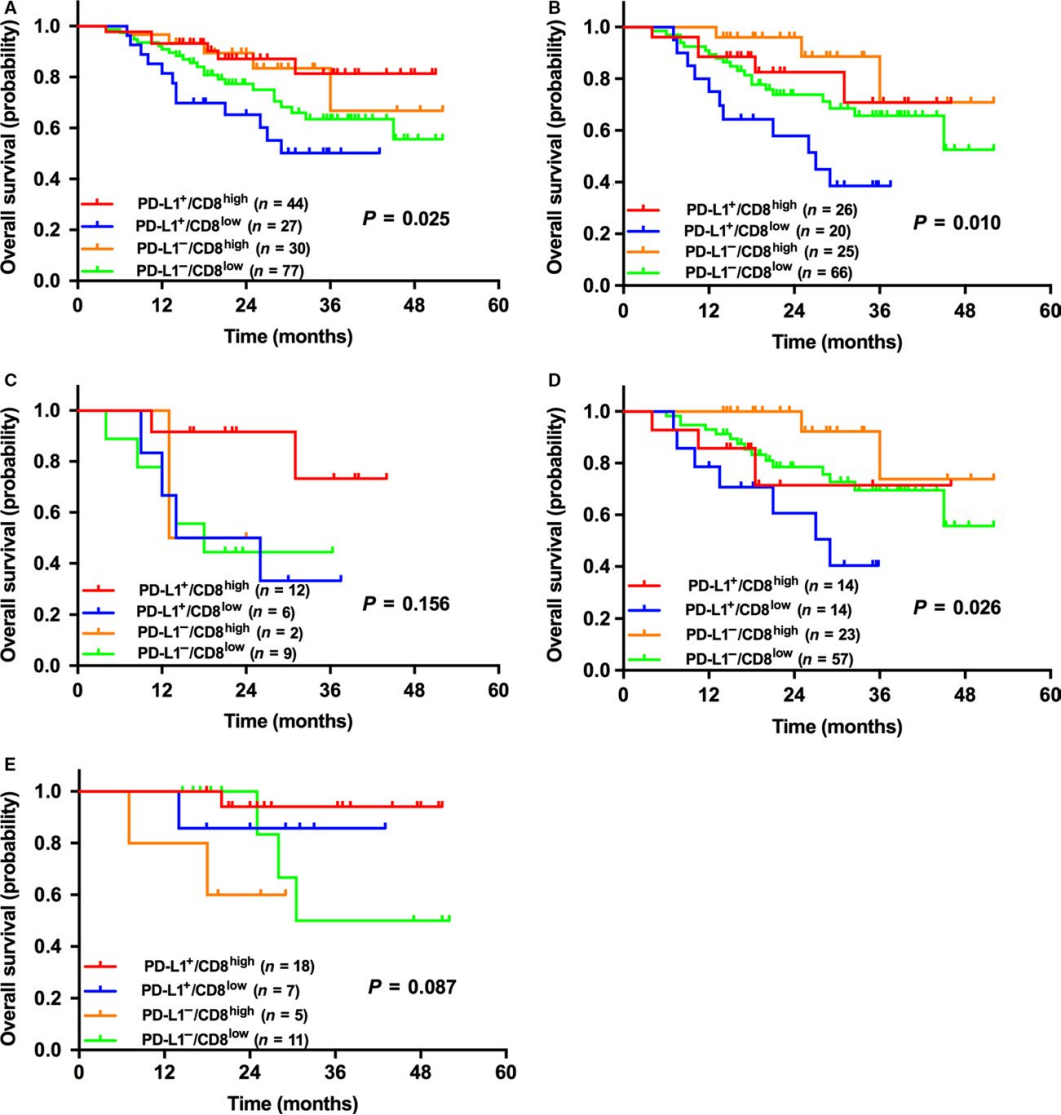
**Kaplan–Meier survival curves of patients with NSCLC (A), ADC (B), solid ADC (C), non‐solid ADC (D), and SqCC (E) sub‐grouped by the combination of PD‐L1 expression (5% cut‐off) and CD8^+^ TILs density (5% cut‐off).** Significant statistical differences were shown in patients with NSCLC, ADC and non‐solid ADC (*P *= 0.025, 0.010 & 0.026, respectively). In patients with ADC and non‐solid ADC, patients with PD‐L1^–^/CD8^high^ had the longest OS, and PD‐L1^+^/CD8^low^ had the shortest OS. However, among the whole NSCLC patients, patients with PD‐L1^+^/CD8^high^ had the longest OS, and PD‐L1^–^/CD8^low^ had the shortest OS. No statistical differences were shown in patients with solid ADC and SqCC.

We divided the patients into four subgroups (PD‐L1^+^/CD8^high^, PD‐L1^+^/CD8^low^, PD‐L1^−^/CD8^high^, and PD‐L1^−^/CD8^low^). Statistical differences were achieved in patients with NSCLC, ADC and non‐solid ADC (*P *=* *0.025, 0.010 & 0.026, respectively), but not in patients with solid ADC and SqCC (*P *=* *0.156 & 0.087, respectively). Among the whole cohort, the longest OS was achieved in patients with PD‐L1^+^/CD8^high^, and the shortest was in PD‐L1^+^/CD8^low^ (median OS was not reached). In groups of ADC and non‐solid ADC, patients with PD‐L1^+^/CD8^low^ experienced the worst OS (median OS: 27.0 months & 29.0 months, respectively), and patients with PD‐L1^−^/CD8^high^ achieved the best OS (median OS was undefined in both groups) (Fig. 3).

### The Correlation between PD‐L1 expression/CD8^+^ TILs density and OS among the patients with diverse ODM status

**Figure 4:**
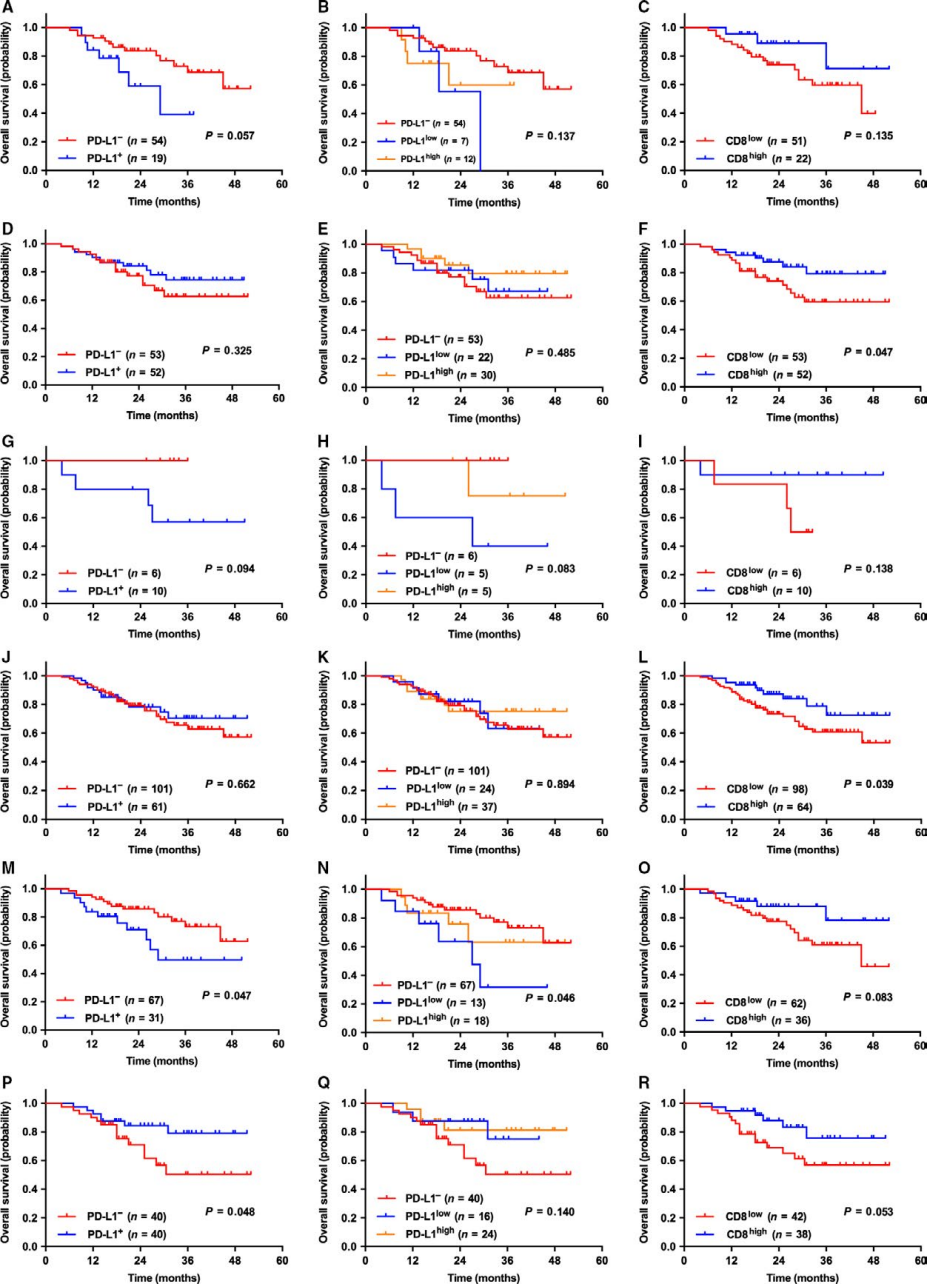
**The correlation between the expression levels of PD‐L1 (on tumor cells) or CD8^+^ TILs density and OS among NSCLC patients with diverse ODM status.** In NSCLC patients with *EGFR* ^+^ (A–C) and *KRAS* ^+^ (G–I), no statistical differences were shown. In NSCLC patients with *EGFR* ^–^ (D–F) and *KRAS* ^–^ (J–L), patients with CD8^high^ had longer OS than those with CD8^low^ (*P *= 0.047 & 0.039, respectively). In patients with ODM^+^ (M–O) and ODM^–^ (P–R), it showed no correlation between CD8^+^ TILs density and OS; PD‐L1 expression (5% cut‐off) was associated with OS in both groups of patients: ODM^+^ patients with PD‐L1^–^ had longer OS (*P *= 0.047), meanwhile, ODM^–^ patients with PD‐L1^+^ had longer OS (*P *= 0.048).

In patients with *EGFR* ^−^ or *KRAS* ^−^, CD8^high^ TILs were associated with better OS (*P *=* *0.047 & 0.039, respectively), but there was no statistically significant difference between PD‐L1 expression levels and OS. In patients with *EGFR* ^+^ and *KRAS* ^+^, PD‐L1 expression levels or CD8^+^ TILs density were not associated with OS. In patients with ODM^+^, it was observed that PD‐L1^−^ was associated with longer OS (*P *=* *0.047); in contrast, PD‐L1^+^ was associated with longer OS in patients with ODM^−^ (*P *=* *0.048), and there was no correlation between CD8^+^ TILs density and OS in both groups of patients (*P *=* *0.083 & 0.053, respectively). It is worth mentioning that whether statistically significant differences were reached or not, CD8^high^ TILs showed a tendency for better OS than did CD8^low^ TILs in each group of patients, and PD‐L1^+^/PD‐L1^−^ showed a tendency for longer OS than did PD‐L1^−^/PD‐L1^+^ in patients with ODM^−^/ODM^+^, respectively (Fig. 4). However, given the number of *KRAS* ^+^ tumors in the series is not quite large, large‐cohort studies are needed to further confirm the related conclusions.

### Expression consistency of PD‐L1/CD8^+^ TILs between primary lesions and corresponding lymph nodes in NSCLC patients and analyses of OS

**Figure 5:**
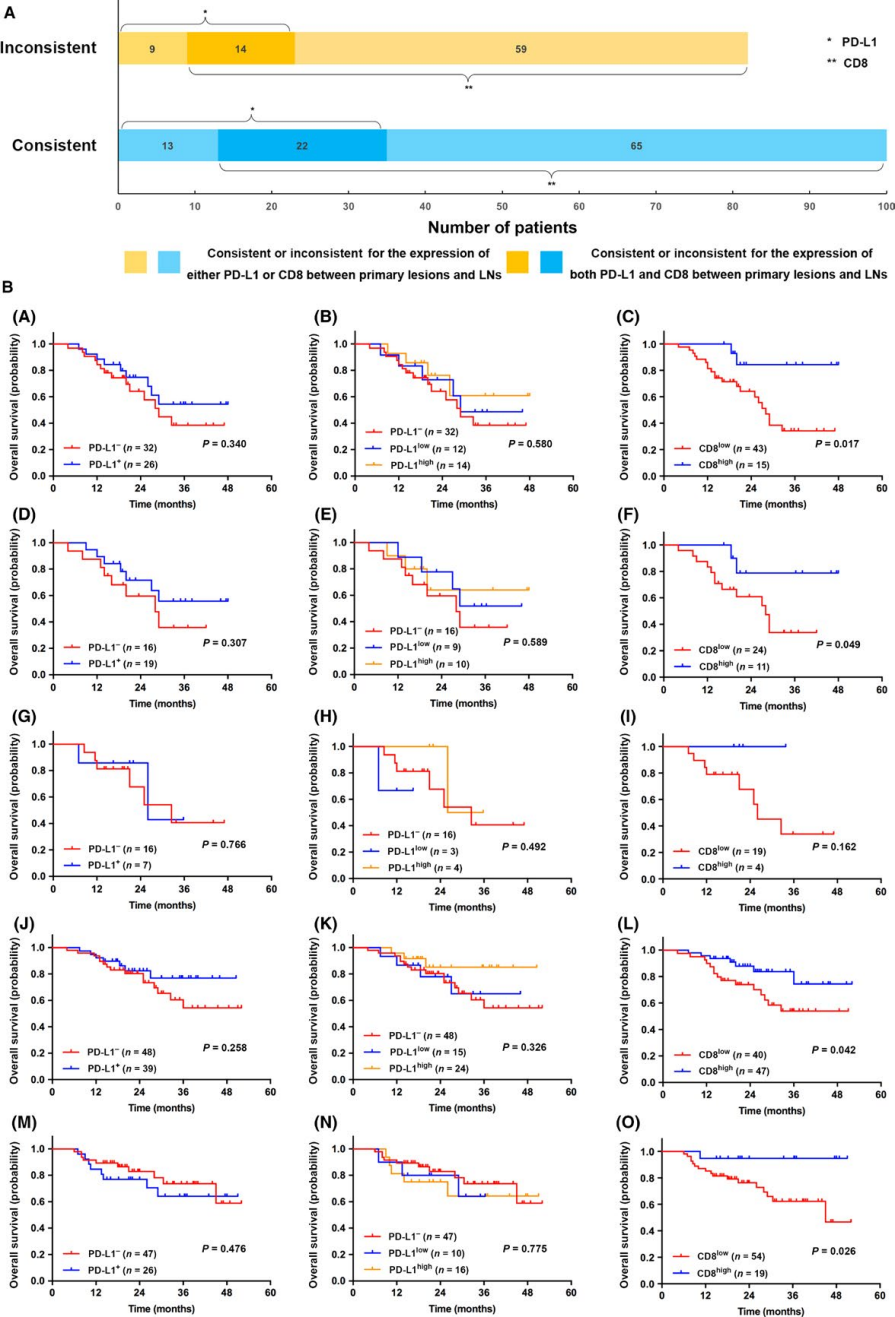
**(A) Bar chart showing the quantity of cases of consistent/inconsistent expression of PD‐L1/CD8^+^ TILs between PLs and LNs; (B) Kaplan–Meier survival curves of patients with LN^+^ (A–C), consistent expression of PD‐L1 (between PLs and LNs) (D–F), inconsistent expression of PD‐L1 (G–I), consistent expression of CD8^+^ TILs density (J–L), inconsistent expression of CD8^+^ TILs density (M–O).** Other than patients with inconsistent expression of PD‐L1, CD8^high^ TILs was associated with better OS than CD8^low^ in the rest four groups. It showed no correlation between PD‐L1 expression and OS among all the five groups. Notes: LN^+^ refers to metastatic lymph nodes (patients staged as p‐TxN1‐3Mx). PLs, primary lesions; LNs, lymph nodes.

In the whole cohort, 58 patients had metastatic lymph nodes, so we assessed the expression consistency of PD‐L1 protein on tumor cells using the 58 pairs of samples. Consistent and inconsistent PD‐L1 expression levels between primary lesions and metastatic lymph nodes were found in 35 and 23 patients, respectively. After performing survival analyses, statistically significant differences between CD8^high^ TILs and longer OS in the 58 patients and patients with consistent PD‐L1 expression (*P *=* *0.017 & 0.049, respectively) were noted, but no correlation between CD8^+^ TILs density and OS in patients with inconsistent PD‐L1 expression (*P *=* *0.162). Due to tissue availability, we assessed the expression consistency of CD8^+^ TILs in 160 patients. Consistent expression was detected in 87 patients. Survival analyses indicated that CD8^high^ TILs were associated with better OS in patients with either consistent or inconsistent expression (*P *=* *0.042 & 0.026, respectively), and just as seen in the results in patients with metastatic lymph nodes, there was no prognostic value of PD‐L1 expression in OS (*P *>* *0.05) (Fig. 5).

## Discussion

Blockade of the PD‐1/PD‐L1 pathway has been the standard second‐line therapy for advanced NSCLC 7, 8, 10. For patients with PD‐L1 positive expression, PD‐1/PD‐L1 inhibitors showed significantly longer PFS and OS than traditional chemotherapy in the first‐line treatment setting 25. However, the prevalence and prognostic value of PD‐L1 expression levels in advanced NSCLC remains controversial, so does that of CD8^+^ TILs 26, 27, 28, 29, 30, 31, 32, 33. In this study, we found PD‐L1^+^ was associated with male, smokers, SqCC and *EGFR* ^−^. CD8^+^ TILs was more common in smokers, SqCC, p‐stage I and *EGFR* ^−^. CD8^+^ TILs, not PD‐L1, was an independent predictive factor for OS.

PD‐L1^+^ was detected in 39.9% patients in our study. Of them, 59.2% (42/71) were strong positive (PD‐L1^high^). PD‐L1^high^ was also associated with SqCC and *EGFR* ^−^ (*P *=* *0.001 & 0.045). The reported extents of PD‐L1 positive expression in NSCLC ranged from 7.4% to 72.7%, which could be explained by the differences in the choice of primary IHC antibody, ethnicity, cut‐off value of positivity, and histologic types 15, 26, 27, 28, 29, 31, 32, 34, 35. Similarly, these studies could not reach an agreement on the clinicopathological features of PD‐L1 expression in NSCLC. A meta‐analysis including 1550 NSCLC patients showed that only poor tumor differentiation was significantly associated with PD‐L1 expression, while smoking history was marginally associated with PD‐L1 expression 36. However, there was no large‐scale study on PD‐L1 expression in NSCLC to validate this conclusion. As we know, resected NSCLC specimens could reflect PD‐L1 and TILs expression levels better than biopsy or tissue microarray 37. Our study, including 178 patients with resected NSCLC, found that PD‐L1^+^ was common in male, smokers, SqCC, and *EGFR* ^−^. Of note, there was a significant linear correlation between male, smokers, SqCC and *EGFR* ^−^ in our study. Therefore, PD‐L1^+^ may be actually associated with one of mentioned‐above parameters.

Our study found CD8^high^ TILs occurred in 41.6% patients and was associated with smokers, SqCC, p‐stage I, and *EGFR* ^−^. The similar clinicopathological features of CD8^high^ TILs with those of PD‐L1^+^ may be explained by the positive correlation between CD8^+^ TILs density and PD‐L1 expression levels 30, 31. In addition, we found CD8^high^ TILs, instead of PD‐L1^+^, was significantly associated with longer OS. CD8^+^ TILs, which form the pivotal component of the cellular immune system, constitutes the effector arm of adaptive immunity against tumor cells. More recent studies found CD8^+^ TILs was associated with better survival in NSCLC although the correlation remains controversial 13, 16, 38. A study included 1290 NSCLC patients showed a significant correlation between CD8^+^ TILs and longer OS only in SqCC, particularly in early stage 39. A recent study also suggested low CD8^+^ TILs were associated with poor survival in non‐ADC 40. Interestingly, we observed a significant correlation between CD8^high^ TILs and longer OS in all NSCLCs except non‐solid ADCs. On the contrary, there was a significant correlation between PD‐L1^+^ and longer OS in non‐solid ADCs and SqCCs. These findings could be explained in part by the possible impact of histological types on the correlation between CD8^+^ TILs/PD‐L1 expression and OS.

We explored the prognostic effects of PD‐L1 combined with CD8^+^ TILs because CD8^+^ TILs could produce IFN‐*γ* and induce PD‐L1 expression in different solid tumors. In all NSCLCs, the longest OS was achieved in PD‐L1^+^/CD8^high^ group while the shortest OS was in PD‐L1^+^/CD8^low^. Tokito et al. found that the PD‐L1^−^/CD8^low^ group had the shortest survival but the PD‐L1^−^/CD8^high^ group had the longest survival in locally advanced NSCLC patients receiving concurrent chemoradiotherapy 13. This discrepancy may be attributed to the differences in stages of disease, specimen types and cut‐off values. PD‐L1 combined with CD8^+^ TILs may be more useful to predict the prognosis of NSCLC and the efficacy of immunotherapy.

PD‐L1^+^ was associated with *EGFR* ^−^, but not with other oncogenic alternations mentioned in our study. We also observed that PD‐L1^+^ was associated with male, smokers, and SqCC. As we know, *EGFR* ^−^ was common in male patients with smoking history and SqCC histology. Moreover, CD8^+^ was also only associated with *EGFR* ^−^. It may be attributed in part to the significant correlation between PD‐L1^+^ and CD8^high^ TILs in our study. The number of studies evaluating the correlation of PD‐L1/CD8^+^ TILs with ODM was limited. Some studies reported a significant association between PD‐L1 expression with *EGFR* ^+^ or *KRAS* ^+^ in NSCLCs, but others did not 26, 35, 41, 42. Several clinical trials reported PD‐L1 expression was associated with *EGFR* ^+^ and *ALK* ^+^ in NSCLC with up to 72% and 78% of PD‐L1 positive rates in *EGFR* ^+^ and *ALK* ^+^ patients, respectively 31, 35, 41, 43. However, the association between PD‐L1 expression and these ODMs was not observed in other studies 44. The PD‐L1 expression levels on tumor cells may be up‐ or down‐regulated by variable antitumor treatments. Therefore, resected NSCLC specimens from patients in early stages may reflect the initial correlation between PD‐L1 expression and ODMs.

The last but not the least, our study showed inconsistent PD‐L1 expression levels between primary lesions and metastatic lymph nodes occurred in 23 of 58 (39.7%) patients. Moreover, there was a significant correlation between CD8^+^ TILs and longer OS in patients with consistent PD‐L1 expressions between primary lesions and metastatic lymph nodes but not in patients with inconsistent PD‐L1 expression. The question is which specimens should we use to detect PD‐L1 expression in advanced NSCLC. The primary lesions or the metastatic lymph nodes? We considered that it may be better to evaluate PD‐L1 expression in both for advanced NSCLC patients. Patients with consistent positive PD‐L1 expressions may obtain better survival benefit from anti‐PD‐1 inhibitors.

Although many studies and clinical series reported the prevalence, clinicopathological and/or molecular features, and prognostic value of PD‐L1 expression in NSCLC, there were conflicting results on these aspects. The above‐described discrepant results could be explained by the differences in ethnicity, specimen types, PD‐L1 IHC protocols (including primary antibodies), scoring criteria, cut‐off values, targeted cell types (tumor cells or TILs), and locations (membranous or cytoplasmic). The absence of a universally accepted PD‐L1 IHC standard and its interpretation make it extremely difficult to determine the prognostic or predictive value of PD‐L1 expression in NSCLC. Besides, the significance of PD‐L1 expression from a single‐biopsy specimen in advanced NSCLC may be overestimated in clinical practice. Therefore, a large‐scale, prospective study is warranted to determine the prevalence and role of PD‐L1 expression, comparison on PD‐L1 expression between single‐biopsies and resected specimens, and the correlation with TILs in NSCLC population.

## Conflict of Interest

The authors have declared no conflicts of interest.

## Supporting information


**Figure S1.** Representative IHC images for PD‐L1 and CD8^+^ TILs in NSCLC.

**Figure S2.** Representative images of positive and negative controls for PD‐L1 IHC staining.

## References

[1] Torre, L. A. , F. Bray , R. L. Siegel , J. Ferlay , J. Lortet‐Tieulent , and A. Jemal . 2015 Global cancer statistics, 2012. CA Cancer J. Clin. 65:87–108. 25651787 10.3322/caac.21262

[2] Reck, M. , D. Rodriguez‐Abreu , A. G. Robinson , R. Hui , T. Csoszi , A. Fulop , et al. 2016 Pembrolizumab versus chemotherapy for PD‐L1‐positive non‐small‐cell lung cancer. N. Engl. J. Med. 375:1823–1833. 27718847 10.1056/NEJMoa1606774

[3] Hegde, P. S. , V. Karanikas , and S. Evers . 2016 The where, the when, and the how of immune monitoring for cancer immunotherapies in the era of checkpoint inhibition. Clin. Cancer Res. 22:1865–1874. 27084740 10.1158/1078-0432.CCR-15-1507

[4] Chen, D. S. , and I. Mellman . 2013 Oncology meets immunology: the cancer‐immunity cycle. Immunity 39:1–10. 23890059 10.1016/j.immuni.2013.07.012

[5] Pardoll, D. M. 2012 The blockade of immune checkpoints in cancer immunotherapy. Nat. Rev. Cancer 12:252–264. 22437870 10.1038/nrc3239 PMC4856023

[6] Postow, M. A. , M. K. Callahan , and J. D. Wolchok . 2015 Immune checkpoint blockade in cancer therapy. J. Clin. Oncol. 33:1974–1982. 25605845 10.1200/JCO.2014.59.4358 PMC4980573

[7] Borghaei, H. , L. Paz‐Ares , L. Horn , D. R. Spigel , M. Steins , N. E. Ready , et al. 2015 Nivolumab versus docetaxel in advanced nonsquamous non‐small‐cell lung cancer. N. Engl. J. Med. 373:1627–1639. 26412456 10.1056/NEJMoa1507643 PMC5705936

[8] Brahmer, J. , K. L. Reckamp , P. Baas , L. Crino , W. E. Eberhardt , E. Poddubskaya , et al. 2015 Nivolumab versus docetaxel in advanced squamous‐cell non‐small‐cell lung cancer. N. Engl. J. Med. 373:123–135. 26028407 10.1056/NEJMoa1504627 PMC4681400

[9] Fehrenbacher, L. , A. Spira , M. Ballinger , M. Kowanetz , J. Vansteenkiste , J. Mazieres , et al. 2016 Atezolizumab versus docetaxel for patients with previously treated non‐small‐cell lung cancer (POPLAR): a multicentre, open‐label, phase 2 randomised controlled trial. Lancet 387:1837–1846. 26970723 10.1016/S0140-6736(16)00587-0

[10] Herbst, R. S. , P. Baas , D. W. Kim , E. Felip , J. L. Perez‐Gracia , J. Y. Han , et al. 2016 Pembrolizumab versus docetaxel for previously treated, PD‐L1‐positive, advanced non‐small‐cell lung cancer (KEYNOTE‐010): a randomised controlled trial. Lancet 387:1540–1550. 26712084 10.1016/S0140-6736(15)01281-7

[11] Herbst, R. S. , J. C. Soria , M. Kowanetz , G. D. Fine , O. Hamid , M. S. Gordon , et al. 2014 Predictive correlates of response to the anti‐PD‐L1 antibody MPDL3280A in cancer patients. Nature 515:563–567. 25428504 10.1038/nature14011 PMC4836193

[12] Teng, M. W. , S. F. Ngiow , A. Ribas , and M. J. Smyth . 2015 Classifying cancers based on T‐cell infiltration and PD‐L1. Cancer Res. 75:2139–2145. 25977340 10.1158/0008-5472.CAN-15-0255 PMC4452411

[13] Tokito, T. , K. Azuma , A. Kawahara , H. Ishii , K. Yamada , N. Matsuo , et al. 2016 Predictive relevance of PD‐L1 expression combined with CD8+ TIL density in stage III non‐small cell lung cancer patients receiving concurrent chemoradiotherapy. Eur. J. Cancer 55:7–14. 26771872 10.1016/j.ejca.2015.11.020

[14] Schmidt, L. H. , A. Kummel , D. Gorlich , M. Mohr , S. Brockling , J. H. Mikesch , et al. 2015 PD‐1 and PD‐L1 expression in NSCLC indicate a favorable prognosis in defined subgroups. PLoS One 10:e0136023. 26313362 10.1371/journal.pone.0136023 PMC4552388

[15] Velcheti, V. , K. A. Schalper , D. E. Carvajal , V. K. Anagnostou , K. N. Syrigos , M. Sznol , et al. 2014 Programmed death ligand‐1 expression in non‐small cell lung cancer. Lab. Invest. 94:107–116. 24217091 10.1038/labinvest.2013.130 PMC6125250

[16] Wakabayashi, O. , K. Yamazaki , S. Oizumi , F. Hommura , I. Kinoshita , S. Ogura , et al. 2003 CD4+ T cells in cancer stroma, not CD8+ T cells in cancer cell nests, are associated with favorable prognosis in human non‐small cell lung cancers. Cancer Sci. 94:1003–1009. 14611679 10.1111/j.1349-7006.2003.tb01392.x PMC11160236

[17] Cheng, N. , W. Cai , S. Ren , X. Li , Q. Wang , H. Pan , et al. 2015 Long non‐coding RNA UCA1 induces non‐T790M acquired resistance to EGFR‐TKIs by activating the AKT/mTOR pathway in EGFR‐mutant non‐small cell lung cancer. Oncotarget 6:23582–23593. 26160838 10.18632/oncotarget.4361 PMC4695138

[18] Taube, J. M. , R. A. Anders , G. D. Young , H. Xu , R. Sharma , T. L. McMiller , et al. 2012 Colocalization of inflammatory response with B7‐h1 expression in human melanocytic lesions supports an adaptive resistance mechanism of immune escape. Sci. Transl. Med. 4:127ra137. 10.1126/scitranslmed.3003689 PMC3568523 22461641

[19] Topalian, S. L. , F. S. Hodi , J. R. Brahmer , S. N. Gettinger , D. C. Smith , D. F. McDermott , et al. 2012 Safety, activity, and immune correlates of anti‐PD‐1 antibody in cancer. N. Engl. J. Med. 366:2443–2454. 22658127 10.1056/NEJMoa1200690 PMC3544539

[20] Cai, W. , C. Su , X. Li , L. Fan , L. Zheng , K. Fei , et al. 2013 KIF5B‐RET fusions in Chinese patients with non‐small cell lung cancer. Cancer 119:1486–1494. 23378251 10.1002/cncr.27940

[21] Ju, L. , M. Han , C. Zhao , and X. Li . 2016 EGFR, KRAS and ROS1 variants coexist in a lung adenocarcinoma patient. Lung Cancer 95:94–97. 27040858 10.1016/j.lungcan.2016.03.005

[22] Li, X. , C. Zhao , C. Su , S. Ren , X. Chen , and C. Zhou . 2016 Epidemiological study of HER‐2 mutations among EGFR wild‐type lung adenocarcinoma patients in China. BMC Cancer 16:828. 27793199 10.1186/s12885-016-2875-z PMC5084329

[23] Ren, S. , X. Chen , P. Kuang , L. Zheng , C. Su , J. Li , et al. 2012 Association of EGFR mutation or ALK rearrangement with expression of DNA repair and synthesis genes in never‐smoker women with pulmonary adenocarcinoma. Cancer 118:5588–5594. 22569898 10.1002/cncr.27603

[24] Topalian, S. L. , C. G. Drake , and D. M. Pardoll . 2015 Immune checkpoint blockade: a common denominator approach to cancer therapy. Cancer Cell 27:450–461. 25858804 10.1016/j.ccell.2015.03.001 PMC4400238

[25] Reck, M. , D. Rodríguez‐Abreu , A. G. Robinson , R. Hui , T. Csoszi , A. Fülöp , et al. 2016 KEYNOTE‐024: Pembrolizumab (pembro) vs platinum‐based chemotherapy (chemo) as first‐line therapy for advanced NSCLC with a PD‐L1 tumor proportion score (TPS) ≥50%. Ann. Oncol. 27:LBA8_PR.

[26] Azuma, K. , K. Ota , A. Kawahara , S. Hattori , E. Iwama , T. Harada , et al. 2014 Association of PD‐L1 overexpression with activating EGFR mutations in surgically resected nonsmall‐cell lung cancer. Ann. Oncol. 25:1935–1940. 25009014 10.1093/annonc/mdu242

[27] Calles, A. , X. Liao , L. M. Sholl , S. J. Rodig , G. J. Freeman , M. Butaney , et al. 2015 Expression of PD‐1 and its ligands, PD‐L1 and PD‐L2, in smokers and never smokers with KRAS‐mutant lung cancer. J. Thorac. Oncol. 10:1726–1735. 26473645 10.1097/JTO.0000000000000687

[28] Cooper, W. A. , T. Tran , R. E. Vilain , J. Madore , C. I. Selinger , M. Kohonen‐Corish , et al. 2015 PD‐L1 expression is a favorable prognostic factor in early stage non‐small cell carcinoma. Lung Cancer 89:181–188. 26024796 10.1016/j.lungcan.2015.05.007

[29] D'Incecco, A. , M. Andreozzi , V. Ludovini , E. Rossi , A. Capodanno , L. Landi , et al. 2015 PD‐1 and PD‐L1 expression in molecularly selected non‐small‐cell lung cancer patients. Br. J. Cancer 112:95–102. 25349974 10.1038/bjc.2014.555 PMC4453606

[30] Kim, M. Y. , J. Koh , S. Kim , H. Go , Y. K. Jeon , and D. H. Chung . 2015 Clinicopathological analysis of PD‐L1 and PD‐L2 expression in pulmonary squamous cell carcinoma: comparison with tumor‐infiltrating T cells and the status of oncogenic drivers. Lung Cancer 88:24–33. 25662388 10.1016/j.lungcan.2015.01.016

[31] Koh, J. , H. Go , B. Keam , M. Y. Kim , S. J. Nam , T. M. Kim , et al. 2015 Clinicopathologic analysis of programmed cell death‐1 and programmed cell death‐ligand 1 and 2 expressions in pulmonary adenocarcinoma: comparison with histology and driver oncogenic alteration status. Mod. Pathol. 28:1154–1166. 26183759 10.1038/modpathol.2015.63

[32] Konishi, J. , K. Yamazaki , M. Azuma , I. Kinoshita , H. Dosaka‐Akita , and M. Nishimura . 2004 B7‐H1 expression on non‐small cell lung cancer cells and its relationship with tumor‐infiltrating lymphocytes and their PD‐1 expression. Clin. Cancer Res. 10:5094–5100. 15297412 10.1158/1078-0432.CCR-04-0428

[33] Yang, C. Y. , M. W. Lin , Y. L. Chang , C. T. Wu , and P. C. Yang . 2014 Programmed cell death‐ligand 1 expression in surgically resected stage I pulmonary adenocarcinoma and its correlation with driver mutations and clinical outcomes. Eur. J. Cancer 50:1361–1369. 24548766 10.1016/j.ejca.2014.01.018

[34] Mao, Y. , W. Li , K. Chen , Y. Xie , Q. Liu , M. Yao , et al. 2015 B7‐H1 and B7‐H3 are independent predictors of poor prognosis in patients with non‐small cell lung cancer. Oncotarget 6:3452–3461. 25609202 10.18632/oncotarget.3097 PMC4413666

[35] Tang, Y. , W. Fang , Y. Zhang , S. Hong , S. Kang , Y. Yan , et al. 2015 The association between PD‐L1 and EGFR status and the prognostic value of PD‐L1 in advanced non‐small cell lung cancer patients treated with EGFR‐TKIs. Oncotarget 6:14209–14219. 25895031 10.18632/oncotarget.3694 PMC4546461

[36] Pan, Z. K. , F. Ye , X. Wu , H. X. An , and J. X. Wu . 2015 Clinicopathological and prognostic significance of programmed cell death ligand1 (PD‐L1) expression in patients with non‐small cell lung cancer: a meta‐analysis. J. Thorac. Dis. 7:462–470. 25922726 10.3978/j.issn.2072-1439.2015.02.13 PMC4387432

[37] Ilie, M. , E. Long‐Mira , C. Bence , C. Butori , S. Lassalle , L. Bouhlel , et al. 2016 Comparative study of the PD‐L1 status between surgically resected specimens and matched biopsies of NSCLC patients reveal major discordances: a potential issue for anti‐PD‐L1 therapeutic strategies. Ann. Oncol. 27:147–153. 26483045 10.1093/annonc/mdv489

[38] Schalper, K. A. , J. Brown , D. Carvajal‐Hausdorf , J. McLaughlin , V. Velcheti , K. N. Syrigos , et al. Objective measurement and clinical significance of TILs in non‐small cell lung cancer. J. Natl. Cancer Inst. 2015;107:dju435. 25650315 10.1093/jnci/dju435 PMC4565530

[39] Ruffini, E. , S. Asioli , P. L. Filosso , P. Lyberis , M. C. Bruna , L. Macri , et al. 2009 Clinical significance of tumor‐infiltrating lymphocytes in lung neoplasms. Ann. Thorac. Surg. 87:365–371; discussion 371‐362. 19161739 10.1016/j.athoracsur.2008.10.067

[40] Kinoshita, T. , R. Muramatsu , T. Fujita , H. Nagumo , T. Sakurai , S. Noji , et al. 2016 Prognostic value of tumor‐infiltrating lymphocytes differs depending on histological type and smoking habit in completely resected non‐small‐cell lung cancer. Ann. Oncol. 27:2117–2123. 27502728 10.1093/annonc/mdw319

[41] Akbay, E. A. , S. Koyama , J. Carretero , A. Altabef , J. H. Tchaicha , C. L. Christensen , et al. 2013 Activation of the PD‐1 pathway contributes to immune escape in EGFR‐driven lung tumors. Cancer Discov. 3:1355–1363. 24078774 10.1158/2159-8290.CD-13-0310 PMC3864135

[42] Garon, E. B. , N. A. Rizvi , R. Hui , N. Leighl , A. S. Balmanoukian , J. P. Eder , et al. 2015 Pembrolizumab for the treatment of non‐small‐cell lung cancer. N. Engl. J. Med. 372:2018–2028. 25891174 10.1056/NEJMoa1501824

[43] Ota, K. , K. Azuma , A. Kawahara , S. Hattori , E. Iwama , J. Tanizaki , et al. 2015 Induction of PD‐L1 expression by the EML4‐ALK oncoprotein and downstream signaling pathways in non‐small cell lung cancer. Clin. Cancer Res. 21:4014–4021. 26019170 10.1158/1078-0432.CCR-15-0016

[44] Zhang, Y. , L. Wang , Y. Li , Y. Pan , R. Wang , H. Hu , et al. 2014 Protein expression of programmed death 1 ligand 1 and ligand 2 independently predict poor prognosis in surgically resected lung adenocarcinoma. Onco Targets Ther. 7:567–573. 24748806 10.2147/OTT.S59959 PMC3990506

