# Supplementary figures and images for: Prognostic value of PD‐L1 expression in combination with CD8+ TILs density in patients with surgically resected non‐small cell lung cancer
Source: Cancer Med. 2017 Nov 23;7(1):32–45. doi: 10.1002/cam4.1243 (PMC5773962; doi:10.1002/cam4.1243)

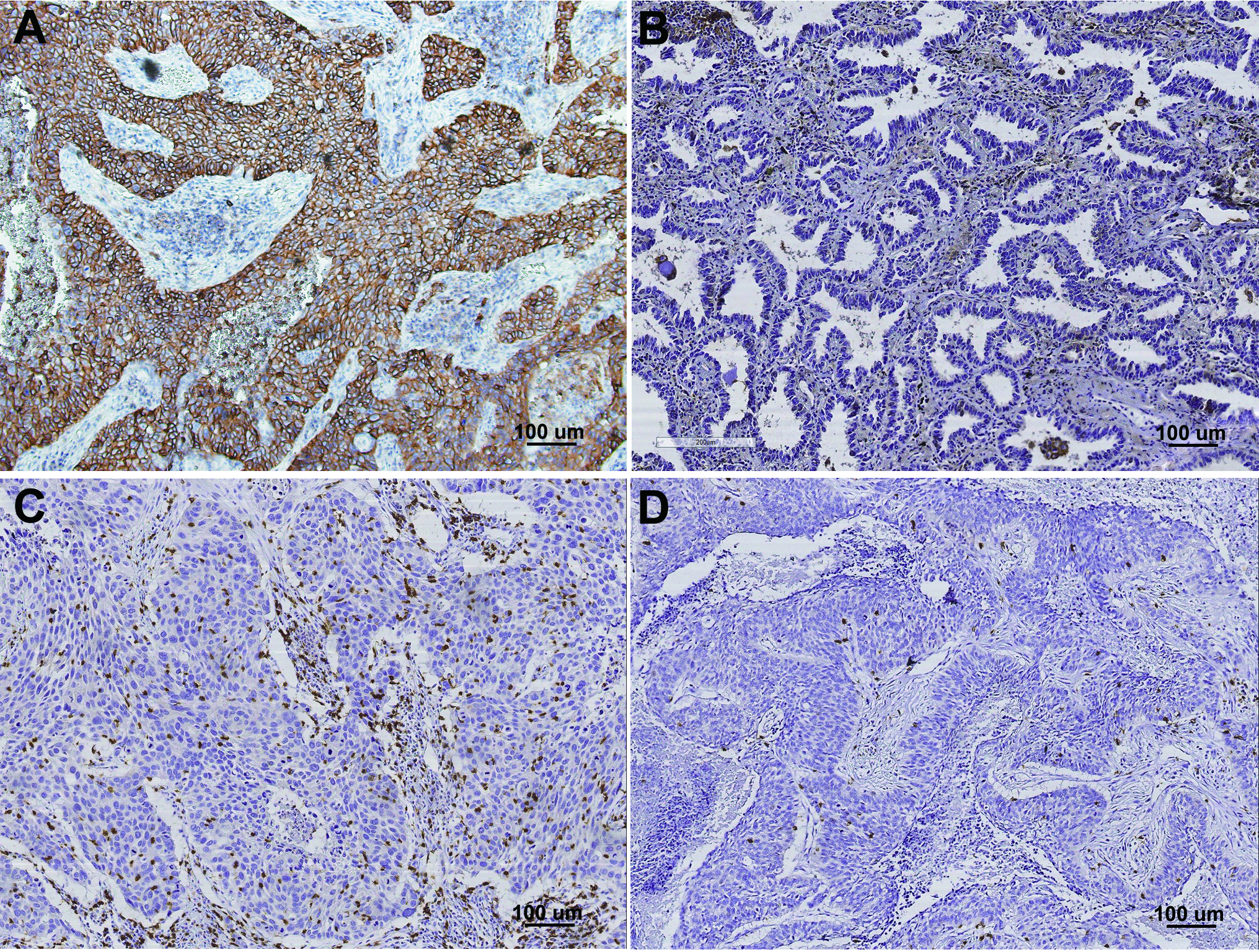

Supplement: Supplementary file 1 — Figure S1. Representative IHC images for PD‐L1 and CD8+ TILs in NSCLC. [file CAM4-7-32-s001.tiff]
